# Eastern Indiana Dental Society

**Published:** 1871-12

**Authors:** J. R. Clayton


### EASTERN INDIANA DENTAL SOCIETY.

In response to a call made by W. C. Stanley, of Dublin, Ind., A. O. Rawls, of Connersville, Ind., J. K. Jameson and J. R. Clayton, Shelbyville, Ind, a meeting was held at the last named place for the purpose of organizing a Local Dental Society, for the mutual improvement and advancement of the interests of the dental profession in Eastern Indiana. Present, W. C. Stanley, A. O. Rawls, J. K. Jameson, John Porter, of Acton, Ind.; B. F. Clayton and J. R. Clayton, of Shelbyville.

On permanent organization, A. O. Rawls was elected President; W. C. Stanley, Vice President; J. K. Jameson, Treasurer, and John R. Clayton, Secretary. The Society to be known as the Eastern Indiana Dental Association.

It was resolved to hold an annual and semi-annual meeting; the annual meetings to be held on the last Tuesday in October, and semi-annual meetings on the first Tuesday in May of each year. The semi-annual meeting will be held at Connersville, Ind., on the first Tuesday in May, 1872, at which time and place it is to be hoped that a good attendance will be given and good interest manifested in behalf of our young Society, which has started out under very favorable auspices

*Executive Committee*—W. C. Stanley, Dublin; B. F. Clayton, J. R. Clayton, Shelbyville, Ind.

J. R. Clayton, *Sec.*

